# Authors’ reply

**DOI:** 10.1192/bjb.2022.49

**Published:** 2022-10

**Authors:** Nagina Khan, Derek K. Tracy

**Affiliations:** Senior research associate in the College of Osteopathic Medicine, Touro University Nevada, Henderson, USA. Email: nkhan786can@gmail.com; Consultant psychiatrist and Medical Director with Oxleas NHS Foundation Trust at Queen Mary's Hospital, London, UK; and a senior lecturer at King's College London and University College London, UK.

We thank Dr Foster for his interest in our work and for taking the time to comment on it. We agree that in a fundamental sense, listening and thoughtful communication have always been at the heart of good clinical care. Our article was written in light of the fact that it remains the case that this does not always happen in practice, and it is a very common refrain from patients and carers that they feel inadequately listened to or involved in care. We thus believe that this problem cannot be over-said or ever presumed to be ‘fixed’. Dr Foster raises two interesting possibilities regarding potential explanations for the lack of completed care plans. These might help explain missing data, although we would note it remains problematic that we do not know whether these are the drivers (and we suspect there will be other causes) and, further, it does not help with the fact that such information should be known and recorded.

